# KiVa-SEND: protocol for a two-arm feasibility cluster randomised controlled trial of an adapted anti-bullying programme for special schools

**DOI:** 10.1186/s40814-026-01813-x

**Published:** 2026-04-11

**Authors:** Julia R. Badger, Lucy Bowes, Christina Salmivalli, Ariel Lindorff, Caitlin Murray, Richard P. Hastings

**Affiliations:** 1 Department of Education, University of Oxford, Oxford, UK; 2 Department of Experimental Psychology, University of Oxford, Oxford, UK; 3 INVEST Research Flagship Centre and Department of Psychology and Speech-Language Pathology, University of Turku, Turku, Finland; 4 School of Policy and Society, University of Birmingham, Birmingham, UK

**Keywords:** SEND, Anti-bullying, KiVa, Intervention, Randomised controlled trial, Children, Young people, Intellectual disabilities

## Abstract

**Background:**

Bullying is a public health risk with rates amongst pupils in mainstream school estimated to be about 20–30%. This increases to approximately 25–69% amongst pupils with special educational needs and disabilities (SEND). Combined bullying data from two large studies of children and young people from 144 countries found that the greatest risk factor to becoming a victim of bullying was being ‘different’ to one’s peers. These differences included factors such as physical appearance, physical disability or learning disability. Yet there are currently no evidence-based anti-bullying programmes designed specifically for pupils in special schools, and therefore no randomised controlled trials. This study adapted KiVa – an established and evidence-based Finnish anti-bullying programme of ten, 1.5 h lessons that can be embedded into a school curriculum – into KiVa-SEND, by adjusting the language, activities and teaching delivery.

**Methods:**

A two-arm feasibility cluster interventional randomised controlled trial with a 1:1 blocked randomisation allocation ratio of schools and an embedded process evaluation. Data will be collected at baseline and at a 12-month follow-up. Eight UK special education schools will participate with between 128 and 384 pupils and between 16 and 96 teachers completing the data questionnaires. A further three to six teachers and up to 10 pupils will participate in the process evaluation interviews or Talking Mats. Talking Mats is a visual tool to support individuals who struggle with communication, to express their thoughts and emotions. Four schools will be allocated to implement KiVa-SEND across the academic year 2025/26 in addition to anti-bullying practice as usual, and four schools will continue with usual practice alone. Primary outcomes will be feasibility outcomes on the topics of recruitment and retention, adherence, staff surveys, pupil surveys and pupil attendance. Secondary outcomes will focus on pupil survey data, teacher survey data, and the differences between KiVa-SEND and the schools’ current anti-bullying programmes. The process evaluation will focus on the topics of recruitment and retention, implementation and adherence of the adapted KiVa programme, engagement and acceptability of/to pupils and staff, and suitability of the outcome measures.

**Discussion:**

This feasibility cluster randomised controlled trial with embedded process evaluation will evaluate the feasibility of delivering KiVa-SEND within a variety of UK special schools, the acceptance of the materials, and the suitability of the outcome measures, for pupils aged 7–14 with a range of primary educational needs and learning disabilities. This will inform the feasibility to later conduct a definitive randomised controlled trial of the effectiveness of KiVa-SEND.

**Trial registration:**

ISRCTN, ISRCTN15516577. Registered 31 March 2025 before any data collection, https://www.isrctn.com/ISRCTN15516577.

## Background

Bullying is a public health risk with rates amongst pupils in mainstream school estimated to be about 20–30%. This increases to approximately 25–69% amongst pupils with special educational needs and disabilities (SEND; [10]), with the higher rates being found for pupils separated from mainstream classes or schools. Bullying involvement (either as a victim, bully, or bully-victim) can have detrimental effects on health, educational and social outcomes [1] with some negative outcomes lasting into adulthood [8]. Although for many children and young people, victimisation occurs at just one or two time points, for some individuals, victimisation persists across time [12]. In 2005, the UK Government acknowledged the complex and significant issue of bullying amongst school-aged children and the following Education and Inspections Act 2006 was enforced which included the legal requirement of every maintained school in England to have a policy in place to prevent all forms of bullying amongst pupils (legislation.gov.uk). An increase in bullying interventions, policies and resources was observed, yet still the UK remains unstandardised in its approach to anti-bullying. Few randomised controlled trials (RCTs) have investigated the effectiveness of any anti-bullying programme or intervention in mainstream schools and in a recent systematic review, it was found that no RCTs have focused on programmes designed specifically for pupils in special schools [3]. Educators in special school contexts may have adapted mainstream school materials, but whether the adapted (or original) materials are suitable or effective with this population has not been evaluated.

Some mainstream school-based anti-bullying interventions have been found to reduce school-level victimisation by, on average, 15–20% with some findings of up to 50% [5]. Programmes such as KiVa [11], a Finnish anti-bullying programme for pupils aged 7–15, are used in mainstream schools, are evidence-based and show effectiveness in reducing victimisation and increasing empathy in UK primary schools [4]. Any pupil can be involved in bullying, yet certain risk factors increase the likelihood of becoming perpetrators and/or victims of bullying. Combined bullying data from two large studies of children and young people from 144 countries found that the greatest risk factor to becoming a victim of bullying was being ‘different’ to one’s peers. These differences included factors such as physical appearance, physical or learning disability, nationality and family-level disadvantage [15, 16]. It is therefore essential to ensure that any resources and programmes used with the most vulnerable pupils such as those with SEND, are appropriate and effective.

The current trial is in response to the lack of understanding and research into anti-bullying programmes for pupils with SEND, a particularly vulnerable population when it comes to bullying involvement. There is a clear lack of evidence-based support, and the need for an RCT to evaluate a potentially suitable intervention. Recently, a preliminary adaptation of two KiVa lessons for use with special school populations was deemed successful with regards to engagement, acceptability and feasibility [2]. The full adaptation is now complete and known as KiVa-SEND.

## Methods

### Aims

The primary aim is to examine the feasibility of the KiVa-SEND anti-bullying programme being delivered successfully by teachers to pupils in special education schools, with a focus on recruitment and retention, implementation and adherence, engagement and acceptability.

The secondary aim is to examine the suitability of potential outcome measures for pupils and staff.

Combined, these will determine the feasibility to later conduct a definitive randomised controlled trial of the effectiveness of KiVa-SEND.

As a feasibility study, no hypotheses are generated. However, the following feasibility outcome data will be collected: Primary outcomes – 1) recruitment and retention, 2) adherence to the programme, 3) staff survey completion, 4) pupil survey completion. Secondary outcomes – 1) pupil self-report data on bullying involvement, 2) staff data on pupil mental health and pupil involvement in bullying, 3) differences in teaching content and action processes to tackle bullying, between all schools’ usual anti-bullying practice, and the KiVa-SEND practice.

### Design

Two-arm feasibility cluster interventional randomised controlled trial with a 1:1 blocked randomisation allocation ratio of schools and an embedded process evaluation. Recruited schools fall within 50 miles from Oxford with baseline data collected in the summer of 2025 and the follow-up data to be collected 12 months later in the summer of 2026, with the KiVa-SEND intervention being delivered throughout the academic year 2025/2026.

### Study setting—schools and participants

Schools: Eight UK special education schools have been recruited. Schools vary in their pupils' primary educational need (including social, emotional & mental health; autism; cognition & learning; moderate learning difficulties) and overall, include a large proportion of pupils with learning disability, either identified from their Education, Health and Care Plan (EHCP) or from the school’s own database. Pupils: Pupils from select classes from year 3 to year 9 (ages 7 – 14) provided baseline data between April – July 2025, and will provide follow-up data between April – July 2026 (when in year 4 – 10, aged 8 – 15). Once schools were recruited, discussions were had with regards to which class groups would participate in the trial. Decisions were based on the age and ability of the pupils to access the KiVa-SEND materials, and the number of classes in the school. Schools were recommended to suggest between 2 and 6 classes to participate. Staff: Data was, and will, also be collected from the teachers at each time point.

Schools opt into the trial, directly to the research team; parents can opt their child out of the research (although in these cases the pupil will still be able to attend the KiVa-SEND lessons if their school is allocated to the intervention), by informing the school, and pupils must tell the researcher they assent to take part in the data collection on the day of data collection. Information letters and opt-out forms were sent out towards the end of March 2025, via the school, to all parents / guardians of eligible pupils. Parents / guardians had a minimum of two weeks to opt their child out of the data collection (although if they did not opt their child out initially but later wish to withdraw their child and any data already collected, this is also possible up to the full anonymisation of the data after the 12-month follow-up).

For the process evaluation, a sub-set of staff and pupils will be invited to take part in interviews (staff and pupils) or a Talking Mats session (pupils). Talking Mats is a visual communication tool that can support individuals who struggle with communication, to express their thoughts and emotions. It uses picture placement on a visual scale (to signify a positive, neutral or negative feeling, for example) about a particular topic of conversation. Separate consent will be required from staff and parents / guardians, and assent must be given by the pupils for these interview sessions.

### Inclusion and exclusion criteria

Schools: Inclusion – 1) special school, 2) within a 50-mile radius of Oxford (to enable our research teams to visit the schools for in-person data collection). Exclusion – 1) mainstream school, 2) opened within the past year (due to small pupil numbers within the school and within class groups, making the school non-representative).

Pupils: Inclusion – 1) attending a special school, 2) aged between 7 and 14 during baseline data collection (for suitability of the programme content). Exclusion—1) pupils who are unable to access the KiVa-SEND materials; for example, those who cannot integrate into a classroom setting or those who cannot access curriculum-style learning, 2) pupils whose parents have opted their child out of data collection, or 3) eligible pupils who joined the school after the baseline data collection.

### Recruitment and retention

The study started 1st October 2024 and will end 30th September 2026. The trial started school recruitment in October 2024 and completed recruitment early March 2025.

The research team created a database of established special schools within a 50-mile radius of Oxford. Schools were recruited via cold calling from the research team, starting with the closest to Oxford and working outwards. Headteachers were informed of all feasibility study processes and school requirements. At the end of the trial, schools will receive a small monetary recompense: the four schools that will be randomly allocated to continue as usual (usual practice) will receive £100 each; the four schools that will be randomly allocated to trial KiVa-SEND with usual practice will receive £275 each in recognition of additional data collection requirements for the research in the intervention arm and for the time taken to adapt their teaching plans to deliver the programme throughout the year. Contact will be kept with all schools throughout the trial to encourage retention.

### Sample size

Data will be collected from 2 – 6 classes per school. With an average of 8 pupils per class, the pupil sample size is 296. With eight schools and a range of 2- 6 classes per school, the teacher sample size is estimated to be between 16 and 96 depending on whether the same teacher is present for the baseline and follow-up data collection. In total, between three and six teachers will participate in the process evaluation interviews, and up to 10 pupils will participate in the process evaluation interviews or Talking Mats. As a feasibility trial to inform a definitive trial we proposed recruiting sufficient schools and pupils to address our feasibility questions, and particularly the question regarding retention of pupils. With a baseline sample size of 296 children and a potential retention rate of 75% (middle of our amber progression thresholds) a 95% CI of 0.05 would be 70%—80% [7]. Recruiting eight schools as stipulated (four in each trial arm), will ensure that even if we experience whole-school drop-out, expected pupil attrition or refusal, or missing pupil or teacher data, we will still be able to conduct our trial with enough data to provide informative findings.

### Study flowchart

**Fig. 1:**
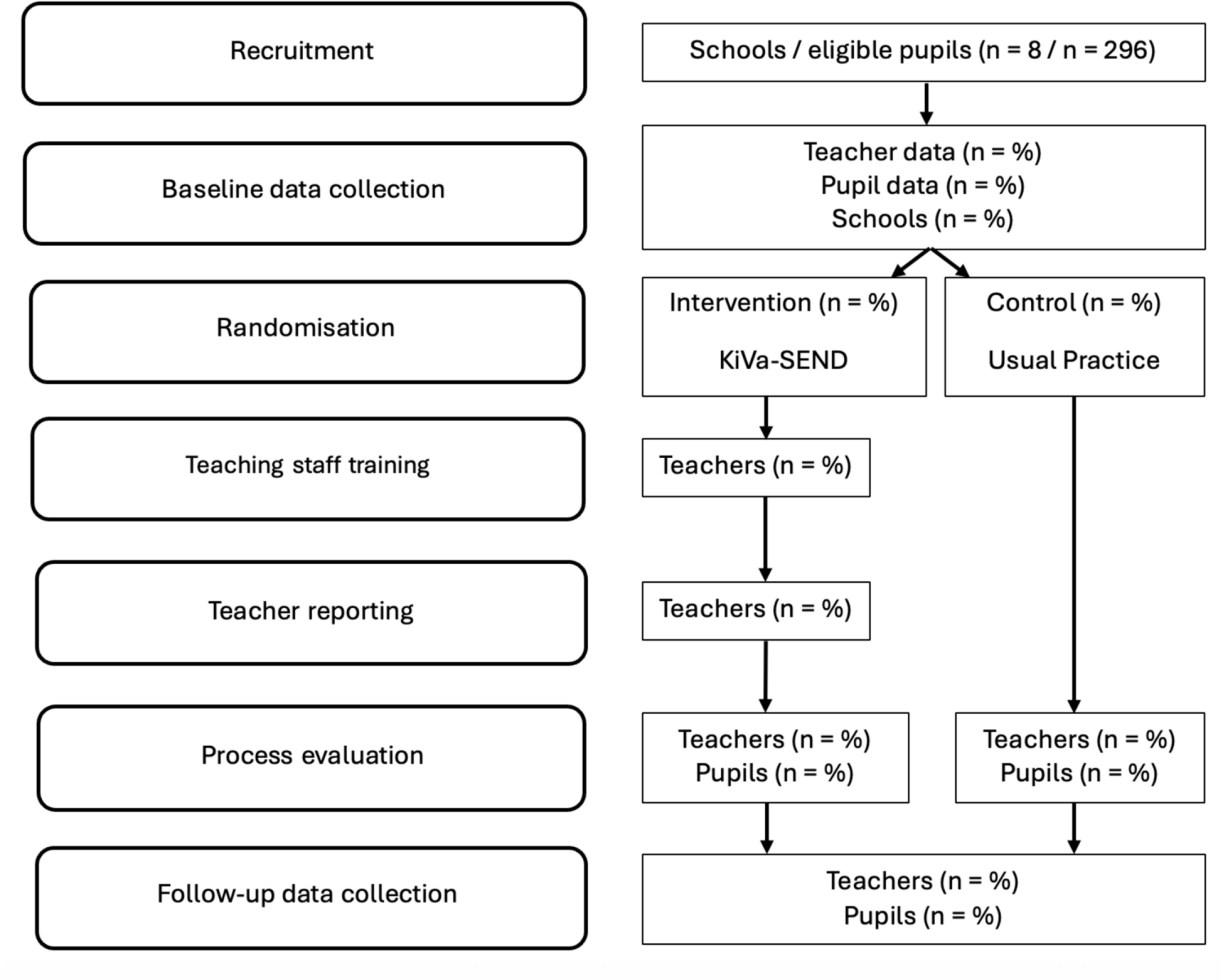
Study flow chart

Figure 1 shows the study flowchart from recruitment through the follow-up data collection.

### Randomisation

The eight schools were randomly allocated using blocked randomisation on a 1:1 basis to either implement KiVa-SEND (intervention group) with usual practice or to continue practice as usual only (comparison group) to understand feasibility of including KiVa-SEND. Randomisation was carried out by one of the trial Co-Investigators (RH) who has no contact with the schools and does not know the school names (codes will be used for the randomisation). The Principal Investigator (JB) informed schools of their allocation once baseline data collection was complete.

The research team randomly allocated the schools to a trial arm (intervention or control), but which classes participated from each school was decided upon by the school.

### Blinding

Schools were informed of their allocation after their baseline data were collected. After allocation, the pupils, teachers and principal investigator were not blind to the intervention status. Although all efforts will be made to keep the majority of the researchers blind to the intervention status, it will be difficult to attend the schools for process evaluation and follow-up data collection and remain blind to the allocation status due to classroom and school displays, and pupils and teachers talking about their KiVa-SEND lessons. We will gather data on unblinding of data collection staff at follow-up to inform the feasibility of researcher blinding in a future trial.

### Intervention: KiVa-SEND

KiVa-SEND is adapted from the anti-bullying programme KiVa. KiVa (Kiusaamista Vastaan: ‘against bullying’) is an evidence-based Finnish anti-bullying programme designed for pupils aged 7–15 [11]. It is currently used in mainstream schools in 23 countries across the world. KiVa has three units, Unit One for ages 7–9, Unit Two for ages 10–12 and Unit Three for ages 13–15 and focuses on delivering and discussing topics such as ‘being part of a group’, ‘school environment’, ‘friendships’, ‘emotions’, ‘inclusion and difference’, as well as traditional bullying and cyberbullying, over the period of one academic year. The KiVa programme focuses on the social architecture of bullying situations and centres on influencing positive pupil behaviours whilst also encouraging bystanders to support victims and stand against bullying.

KiVa is made up of two components: universal actions and indicated actions. Universal actions are found at the class level and school level and are taught and encouraged through a fully manualised set of lessons. Indicated actions are scripted strategies for school staff who form the KiVa Team to manage, document and monitor confirmed cases of bullying involvement.

KiVa-SEND (unpublished) is a UK adaptation and extension of KiVa, which has adapted the materials for use in special schools and for pupils with SEND. Whilst the majority of the lessons are based on the original programme, a few additional lessons have been included to better represent the needs of pupils and staff in special schools. In 2023, the first two adapted lessons were co-developed and successfully piloted in two UK special schools [2]. KiVa-SEND is adapted from KiVa Unit One but also includes the cyberbullying components from Unit Two. The adaptation has simplified the language, avoided abstract concepts, and has increased the quantity and variety of interactive activities to cater to a range of learning styles and abilities. It includes new presentation slides to accompany the teaching of every lesson. Many of the lessons do not require a good level of expressive language skill.

KiVa-SEND will be delivered over one academic year (September 2025 – July 2026) and is designed to fit within each school’s Personal, Social, Health and Economic (PSHE) education classes (a total of 20, 45-min lessons). The four schools randomly allocated to deliver KiVa-SEND were provided with training for two or three members of their staff before delivering the programme. They will then be expected to lead the wider school implementation. Adherence to the intervention will be monitored by the research team via end-of-lesson teacher reporting forms which will reflect on which lesson activities were covered, and the reason for non-coverage.

### Comparison

Comparison schools will complete the baseline and follow-up data collection but will otherwise continue as usual throughout the academic year 2025–2026. Schools (both comparison and intervention) will be asked to provide usual practice data so the researchers can see whether any new strategies were implemented throughout the year and whether implementing KiVa-SEND changed usual practice in intervention schools.

### Intervention discontinuation or modification

Wherever possible, all participants will remain in the trial for baseline and follow-up data collection. However, any school or individual participant can withdraw from the trial by informing the research team. Modifications to KiVa-SEND can be made where deemed appropriate by the class teacher delivering the materials. In this instance, they should report the modifications on the end-of-lesson teacher report.

### Data collection

#### Pupils

Data collection with the pupils (summer 2025 and summer 2026) will use an adapted version of the Olweus Bully/Victim Questionnaire (OBVQ; [9]). This version includes 20 of the original questions about either being a victim of bullying (10 questions) or bullying others (10 questions). Two new questions have been included which ask whether 1) the pupil has *ever* been bullied and 2) has *ever* bullied another pupil. These questions reflect that often pupils in special schools who have been involved in bullying will struggle to separate the incident with a time restraint, for example, within the past three months. These two additional questions allow the pupils to answer more broadly, before being asked to focus in on a limited time window. Based on previous piloting with pupils from three additional special schools, adapted answer options will also be provided which simplify the original OBVQ answer options. Adapted answer options: 1) ‘this has never happened to me’, 2) ‘this hasn’t happened to me since I started in this year group’, 3) ‘this has happened two or three times since I started in this year group’, 4) ‘this has happened to me lots of times since I started in this year group’, 5) ‘I don’t want to say’. Data has, and will be collected on a 1:1 (researcher:pupil) basis unless the school deems small group data collection possible (1:2 or 1:3). Data collection takes approximately 10 min per pupil and is preferably conducted in a quiet room near the pupil’s classroom, or where necessary, in a quiet area within the pupil’s classroom. In some cases, pupils may have an accompanying adult assigned to support them in school. In these instances, this adult joins them when completing the questionnaire with the researcher. The adult supports these pupils to stay focused and to answer the questions, as they do during usual classroom time, but does not reply on behalf of the pupil and where possible, does not look at which answer the pupils selects. All data collection is via electronic tablets with the researcher reading every question-and-answer option out loud, and where needed, pointing to the accompanying text on the pupil’s screen. Where possible, the pupil makes their selection on the tablet whilst the researcher (and any other pupils or adults) look away. Allowing the child to select an answer on their own provided tablet encourages the child to provide an honest answer. Once an answer has been selected, that question disappears and the next appears.

#### Staff

Data collection with teachers of the selected pupils will involve completing two questionnaires in the summer of 2025 and in the summer of 2026: 1) the Teacher Strengths and Difficulties Questionnaire (TSDQ; [6]), a 25-item behavioural screening questionnaire and, 2) an adapted version of the Teachers’ Perception on Bullying questionnaire (TPB [14],), adapted to be answered specifically for each individual pupil. It involves six questions about the pupil being a victim of bullying and six questions about the pupil engaging in bullying others. Questions will be answered using a 5-point Likert scale from ‘hasn’t happened’ to ‘several times a week’, with an extra option to select ‘unsure’. Questionnaires will be printed out on paper and given to the teachers in class packs (one questionnaire per pupil participating). Data collection will take approximately 2 min per pupil questionnaire set, but teachers will have 2–3 days to complete the questionnaires. Teachers assigned to the KiVa-SEND arm of the trial will deliver the 20 KiVa-SEND lessons and for each lesson will note pupil absenteeism. They will also complete a short check sheet on material covered in that lesson (adherence) and suitability of the content (feasibility).

#### School

Data will be collected electronically via secure transfer, from all eight schools. The following data will be provided for every participating pupil: age, sex, class, teacher, primary educational need (using the English school census 13 SEND options) and whether they have a learning disability (moderate, severe, profound and multiple). The schools will also electronically provide their current bullying and behaviour policies and will answer questions on their current anti-bullying programmes.

#### Researchers

Field notes will be collected on paper during data collection in summer 2025 and summer 2026 and transferred to an electronic notes file. Notes will include whether data was collected 1:1 or in a small group (two to three pupils), where data was collected (in the classroom or in a private space), whether an accompanying adult was present (and their level of involvement), level of pupil comprehension (none, limited, good), whether any adaptations had to be made to the questionnaire (such as text size increase or use of symbols), time taken, and another note of relevance.

#### Process evaluation

After two terms (end of spring 2026), in person or remote interviews will be carried out with three to six members of staff (semi-structured interviews of approximately 20 min each). In person conversations will also be carried out with up to 10 pupils (semi-structured interviews or Talking Mats for approximately 10 min each). The split will be one to two teachers from one to two usual practice schools and two to four teachers from two to four interventions schools and all pupils will be from intervention schools. The process evaluation will assess 1) recruitment and retention, 2) implementation and adherence of the adapted KiVa-SEND programme, 3) enjoyment, engagement and acceptability of/to pupils and staff, 4) suitability of the outcome measures (Table 1).

**Table 1:** Schedule of enrolment, data collection, and intervention

	Study period				
	Enrolment	Baseline	Allocation	Intervention phase	12-month follow-up
Timepoint	0	*t*^1^	*t*^2^	*t*^3^	*t*^4^
Eligibility	X				
School opt-in	X				
Parents opt-out	X				
Pupil assent		X			X
Allocation			X		
Pupil demographics		X			
KiVa-SEND				X	
Pupil Olweus Bully/Victim Questionnaire		X			X
Staff Teacher Strengths and Difficulties Questionnaire		X			X
Staff teachers’ perception on bullying		X			X
Teacher reporting				X	
Process evaluation				X	
Lesson fidelity				X	
Pupil attendance				X	

### Participant timeline

#### Data analysis

As a feasibility trial, most of our outcome data will be descriptive. Missing data will be reported but no imputation will take place. Data will be summarised at baseline and follow-up by trial arm. Exploratory analysis on primary and secondary outcome measures (OBVQ, TSDQ and TPB) will be conducted between trial arms at the follow-up time point, with the baseline as a covariate, whilst considering school clustering. Although we are not testing the effectiveness of the KiVa-SEND programme during this feasibility trial, we shall explore the pupil and teacher survey data descriptively and statistically, to ensure they are viable options to use in a full trial or whether they do not appear appropriate for this cohort.

#### Feasibility outcomes

Recruitment and retention – 1) What proportion of approached schools decided to take part in the trial? 2) What proportion of recruited schools remained in the trial? 3) What proportion of eligible pupils are opted out of the trial by parents/guardians? 4) Are schools willing to be randomly assigned (Yes/No)? These questions will be answered using our ongoing school recruitment and retention tracking data.

Fidelity – 1) Are teachers able to implement and adhere to the KiVa-SEND programme with a good level of fidelity (data on percentage adherence to lesson plans)? 2) Are the manualised lesson plans and activities acceptable to the teaching staff (Yes/No – with suggested changes)? These questions will be answered using the post-lesson teacher reporting data.

Staff surveys – Are staff willing and able to complete the outcomes measures (perceived bullying of each pupil; mental health of each pupil) (Yes/No)? This question will be answered based on the number of returned questionnaires.

Pupils – Are pupils able to understand and complete the outcome measures (experience of bullying involvement) (Yes/No)? This question will be answered based on the number of returned questionnaires and on feedback from researchers supporting the completion of pupil questionnaires.

Adherence/attendance – What was the range of pupil attendance to all of the KiVa-SEND lessons. This question will be answered using our post-lesson teacher reporting data on pupil attendance.

Anti-bullying programmes – How is the usual practice of anti-bullying programmes and procedures different from KiVa-SEND? This question will be answered using school anti-bullying policies and data gathered from the school.

### Process evaluation

The project will follow MRC guidelines on process evaluations for complex interventions [13] to explore a) recruitment and retention, b) implementation and adherence of the adapted KiVa programme, c) engagement and acceptability of/to pupils and staff, and d) suitability of the outcome measures. This will help us to shape the research design and methods for a definitive trial. Interviews will be audio-recorded, transcribed, and thematically analysed.

Teaching staff – Three to six semi-structured interviews will be conducted with teaching staff: one to two from one to two comparison schools and two to four from two to four intervention schools. Interviews will build on the tracking data and post-lesson teacher survey data to explore in more depth the school experience of sign-up to the study, their thoughts on supporting school retention, acceptability of the research design and adherence to materials.

Pupils – Up to 10 semi-structured interviews or Talking Mats sessions will be conducted with pupils from interventions schools. Sessions will explore enjoyment and engagement of the lessons, materials, activities and questionnaires.

Framework analysis will be applied to the interview and focus group data, informed by the feasibility questions and the MRC process evaluation framework.

### Data management, security, and confidentiality

Data entry, coding, security, storage and confidentiality will be ensured, in line with GDPR. Only the trial team will have access to the final study dataset.

All pupil and staff names and school names will be stored securely in password protected documents on the researchers’ secure University of Oxford Nexus 365 SharePoint, separate from any trial data. Pupil questionnaire responses will be collected electronically on trial tablets, via the offline Qualtrics application. Once back at the University, the tablets will be connected securely to the Internet and the offline Qualtrics application will be become online, and the data file will be exported to the researchers’ secure University of Oxford Nexus 365 SharePoint. All data will be pseudonymised, inputted with pupil codes instead of names. Paper versions will be available as back-ups in case of technical issues or pupil preference or need. Teacher questionnaire responses will be collected on paper. All paper data copies will be transferred to the University and stored securely in locked cupboards before being entered into the electronic database. Once data has been entered and checked, the original paper copies will be securely shredded.

Interview and Talking Mats audio recordings will be recorded on audio-recorders. The files will be transferred to the researchers’ secure University of Oxford Nexus 365 SharePoint before being transcribed. Once transcribed, anonymised and checked, the original audio files will be deleted. Framework analysis will be used for the analysis of these qualitative data sets.

All data will be password protected and kept secure. The linkage list (linking pupil name with pupil ID) is required to match summer 2025 and summer 2026 data. This list will be password protected and kept separately to all other collected data (which will be anonymous).

No formal data monitoring committee (DMC) is required due to the nature of this feasibility trial. Instead, the trial team will ensure data monitoring and quality assurance.

### Progression criteria

The assessment of whether this feasibility study can later progress to a full definitive study will be determined by several criteria. Progression thresholds of these criteria are set out in Table 2 and following these categorisations: 1) Green: proceed to a full study without modification, 2) Amber: proceed to a full study with caution and some modification, 3) Red: unsuitable to proceed to a full study.

**Table 2:** Progression thresholds to determine suitability of the KiVa-SEND study to progress to a later full definitive study

	Green	Amber	Red
Recruitment of approached schools	≥ 60%	40–59%	≤ 39%
Eligible pupils recruited *	≥ 80%	60–79%	≤ 59%
Baseline pupil data collected	≥ 80%	60–79%	≤ 59%
Baseline teacher data collected	≥ 90%	70–89%	≤ 69%
Number of classes reaching adherence to the KiVa-SEND programme†	≥ 90%	70–89%	≤ 69%
Number of classes reaching delivery fidelity of KiVa-SEND components‡	≥ 80%	60–79%	≤ 59%
Retention of schools at 12-month follow-up	≥ 90%	70–89%	≤ 69%
Follow-up pupil data collected of those still attending the school	≥ 80%	60–79%	≤ 59%
Follow-up teacher data collected for pupils still attending the school	≥ 90%	70–89%	≤ 69%

^*^Eligible pupils are those within the specific classes pre-selected by the school to participate in the KiVa-SEND study. Recruitment of these pupils is determined by whether their parent / guardian opted them out of participating

^†^Adherence is based on at least 70% of lessons (14/20) being delivered to each participating class (max = 20 lessons over one academic year)

^‡^Fidelity is based on at least 80% of lesson components being delivered per lesson to each participating class (components vary between three and six per lesson)

Other data will be used to inform whether progression to a full definitive randomised controlled trial is feasible, including.

- Recruitment and retention, as identified in Table 2, but also process evaluation data will be examined for specific details such as barriers and facilitating factors.
- Adherence and delivery (implementation) as identified in Table 2, but also post-lesson teacher reporting data and process evaluation data will be examined for specific details such as barriers and facilitating factors at the teacher and at the pupil level.
- Engagement and acceptability of the programme, post-lesson teacher reporting data and process evaluation data will be examined for specific details such as barriers and facilitating factors at the teacher and at the pupil level.
- Suitability of potential outcome measures for pupils and staff based on process evaluation data and researcher field notes of pupil comprehension, examined for specific details such as barriers and facilitating factors.

In all cases, recommendations will be incorporated into a future full study protocol.

### Benefits and risks

There are minimal risks of the trial and potentially no immediate benefits. It is possible that some pupils may become upset during the data collection on bullying experience, or during the KiVa-SEND lessons, but researchers and teaching staff will always be around to manage and support these situations if they do arise. School safeguarding procedures will be followed, and our research team’s data collection protocol will also be followed in the case of adverse events. It is possible that bullying perpetration and/or bullying victimisation will reduce for those delivering KiVa-SEND (but this study is not aimed to measure that impact). The trial will lead to a better understanding of the feasibility of running KiVa-SEND in special schools, and the potential to begin a definitive randomised controlled trial to test the effectiveness of the programme.

## Discussion

This two-arm feasibility cluster interventional randomised controlled trial with a 1:1 blocked randomisation allocation ratio of eight UK special schools and an embedded process evaluation will evaluate the feasibility of delivering KiVa-SEND – adaptation of the evidence-based Finnish anti-bullying programme KiVa – whilst recognising that generalisability will be limited in relation to pupils with more complex or profound special educational needs. The adherence, fidelity and acceptance of the materials, engagement of teachers and pupils, and the suitability of the outcome measures will be explored. Although this trial will not be testing the effectiveness of the KiVa-SEND programme to reduce bullying and victimisation, it will help to inform whether the questionnaires used to measure these outcomes are suitable for this population. This manuscript details the proposed feasibility study design and the progression criteria required to move to a full definitive study. Any amendments will be included in future publications.

### Publication and dissemination plan

The full feasibility trial results will be published in 2027 once the trial is complete; any protocol modifications will be communicated. Overall and anonymised results will be shared with the schools, who can share with interested parents / guardians. The results will be presented during research talks and conferences.

## Data Availability

This manuscript has no associated data.
